# Vaccination and Homœopathy

**Published:** 1892-11

**Authors:** Harlyn Hitchcock

**Affiliations:** New York, N. Y.


### VACCINATION AND HOMŒOPATHY.

Harlyn Hitchcock, M. D., New York, N. Y.

What is vaccination?

It is the introduction of the crude morbific products of disease into the tissue of the healthy organism.

What is the object of vaccination?

It is supposed to prevent the development or mitigate the severity of a disease which might possibly affect the individual at some future period.

What is the result of vaccination?

It makes the subject sick.

These are the three fundamental questions involved in the subject of vaccination with the unequivocating answers. The answers consist of one supposition and two facts. The facts are irrefutable. The supposition embraces an indefinite number of theoretical propositions, none of which have been proved to be in accord with the facts as shown by the records of a century of experiment. Hence ignoring the theories and doctrines, the hypothetical matter, homœopathics has only to deal with the facts, viz., what it is, and its results.

Hahnemann said: "The physician's highest and only calling is to make sick people well, which is called healing." Nowhere do we find any admonition to make people sick, nor is there the slightest suggestion that it is advisable to produce artificially a sickness that may or may not occur naturally at some period more or less remote. Our purpose is to remove, not produce sickness. Would it not be absurd to give a person a dose of Colocynth to-day on the supposition that he might have a belly-ache next year? Who would think of placing a splint on one's leg for fear it might be broken at some future time? Yet this is the logic of the vaccination fiend.

Hahnemann recommended the use of remedial measures *when indicated*, not on the supposition that they might be indicated sometime within the next twenty-five years.

Hahnemann recommended the use of remedial agents in the minimum dose and in a potentiated form. He did not approve of the use of crude materials forced into the tissues any more than he approved of their use by the mouth. Nowhere do we find his approval of the use of the crude morbific products of disease, the *débris* and excreta of ulcerous sores, projected into the organism, and if he does not disapprove specifically of any particular proceeding, is that any excuse for indulging in practice which is inconsistent with the general principles of Homœopathy?

This Association has already made emphatic announcement of its allegiance to Hahnemannian principles and has distinctly expressed itself in regard to the empirical use of disease products, even when subjected to the process of potentiation. Is it not, therefore, desirable that the expression of the Association's opinion of the practice of vaccination be as distinctly and decisively expressed?

Vaccine lymph is the product of disease; it is a crude material; it has not been proved as required; it is not administered in the minimum dose and potentiated form; the practice is purely empirical; it makes the subject sick; it produces injury and death; it is not in accord with the law of similars.

Therefore I propose the following as a standing resolution:

*Resolved*, That vaccination with the crude virus is in practice and theory contrary to the laws of Homœopathy, and without fact or logic for its support, and the practice is hereby condemned.

*Resolved*, That compulsory vaccination is unjustifiable and contrary to the rights and privileges of the people.

With reference to the question of compulsory vaccination and its legal status, I take pleasure in presenting the opinion of Judge Thomas M. Wyatt, of New York, which was prepared at my request, and which is appended hereto as a part of this paper.

